# Cutaneous *Paraconiothyrium cyclothyrioides* Infection in Lung Transplant Recipient, Georgia, USA

**DOI:** 10.3201/eid3203.251042

**Published:** 2026-03

**Authors:** Carolyn Mackey, Stephanie Thomas, Lucy S. Witt, Elizabeth Sajewski, Shawn R. Lockhart, Stephanie M. Pouch, Justin Cheeley, Jamie B. MacKelfresh, Jeremy A.W. Gold

**Affiliations:** Georgia Emerging Infections Program, Atlanta, Georgia, USA (C. Mackey, S. Thomas, L.S. Witt, S.M. Pouch); Atlanta Veterans Affairs Health System, Decatur, Georgia, USA (C. Mackey, S. Thomas, L.S. Witt, S.M. Pouch); Emory University School of Medicine, Atlanta (C. Mackey, S. Thomas, L.S. Witt, S.M. Pouch, J. Cheeley, J.B. MacKelfresh); Centers for Diseases Control and Prevention, Atlanta (E. Sajewski, S.R. Lockhart, J.A.W. Gold)

**Keywords:** fungi, *Paraconiothyrium cyclothyrioides*, lung transplantation, antifungal agents, drug interactions, immunocompromised host, fungal infections, molds, infectious skin diseases, surveillance, United States

## Abstract

We report a cutaneous infection caused by *Paraconiothyrium cyclothyrioides*, a rare environmental mold, in a lung transplant recipient in Georgia, USA. The infection resolved with posaconazole after substantial diagnostic delays and related side effects. This case underscores the need for improved clinical awareness, diagnostic testing, treatments, and surveillance for such infections.

Environmentally acquired infections caused by rare molds are a growing public health concern because of potential disease severity, rising numbers of susceptible immunocompromised persons, and changes to the natural environment that are shifting fungal disease epidemiology (*1*,*2*). *Paraconiothyrium cyclothyrioides*, a Coelomycete, is an environmental mold found on plants and in soil (*3*). Seven cases of human *P. cyclothyrioides* infection have been reported, most cutaneous (*3*–*9*). The incidence of *P. cyclothyrioides* and other rare mold infections might be underestimated, however, because routine laboratory methods often cannot identify them or classify them as contaminants. Reliable identification usually requires molecular diagnostics that are not commonly performed. Thus, data to inform clinical recognition, treatment, and future surveillance efforts are sparse. We present a case of cutaneous *P. cyclothyrioides* infection in a lung transplant recipient in Georgia, USA, identified through Centers for Disease Control and Prevention’s Emerging Infections Program (EIP) invasive mold diseases surveillance system.

The EIP is a collaborative effort among public health agencies, healthcare providers, and academic institutions aimed at enhancing public health capacity to address emerging infectious threats (https://www.cdc.gov/emerging-infections-program/php/about/index.html). The EIP invasive mold diseases team in Georgia conducts active, sentinel, laboratory-based surveillance at Atlanta-area healthcare facilities using a standardized case report form to collect patients’ data from electronic medical records. Georgia EIP surveillance activities were approved by the Institutional Review Board, granting a consent and HIPAA waiver.

A 67-year-old man underwent unilateral lung transplantation for idiopathic pulmonary fibrosis. He received basiliximab for induction immunosuppression and a regimen of mycophenolate mofetil, tacrolimus, and prednisone for maintenance immunosuppression. At time of transplantation, after isolation of *Aspergillus niger* from the donor airway, physicians treated the patient with inhaled amphotericin B and oral posaconazole. The patient experienced acute cellular rejection ≈9 months posttransplantation, which physicians treated with thymoglobulin. Thereafter, the patient completed a 16-month course of posaconazole, ending treatment because of fatigue. Physicians identified environmental molds *Penicillium* and *Fonsecaea* species in separate bronchoalveolar lavage cultures shortly after the patient completed posaconazole treatment but did not administer treatment, considering both to be contaminants.

Twenty-eight months after the transplant (1 year after completing posaconazole), the patient noticed a nodule on his shin. He visited a dermatology clinic 4 months later for 2 verrucous pink nodules on his right shin. Skin biopsies done for histopathology and culture showed polymorphic dematiaceous hyphal elements not further speciated, and clinicians initiated no treatment at that first visit. The patient gained referral to an academic dermatology clinic and sought treatment there 3 months later. His lesions had not substantially spread or evolved (Figure 1). Clinicians submitted another tissue biopsy for culture and histopathology. Histopathologic examination revealed numerous polymorphic forms within the superficial dermis, with overlying epidermal pseudoepitheliomatous hyperplasia (Figure 2).

**Figure 1:**
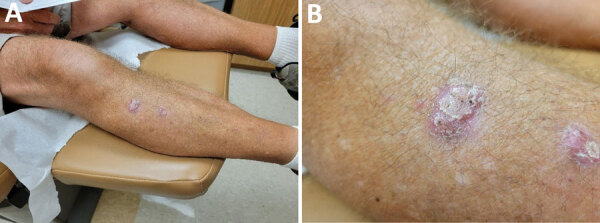
Scaly and crusted pink verrucous nodules clustered on right shin of lung transplant recipient with cutaneous *Paraconiothyrium cyclothyrioides* infection, Georgia, USA.

**Figure 2:**
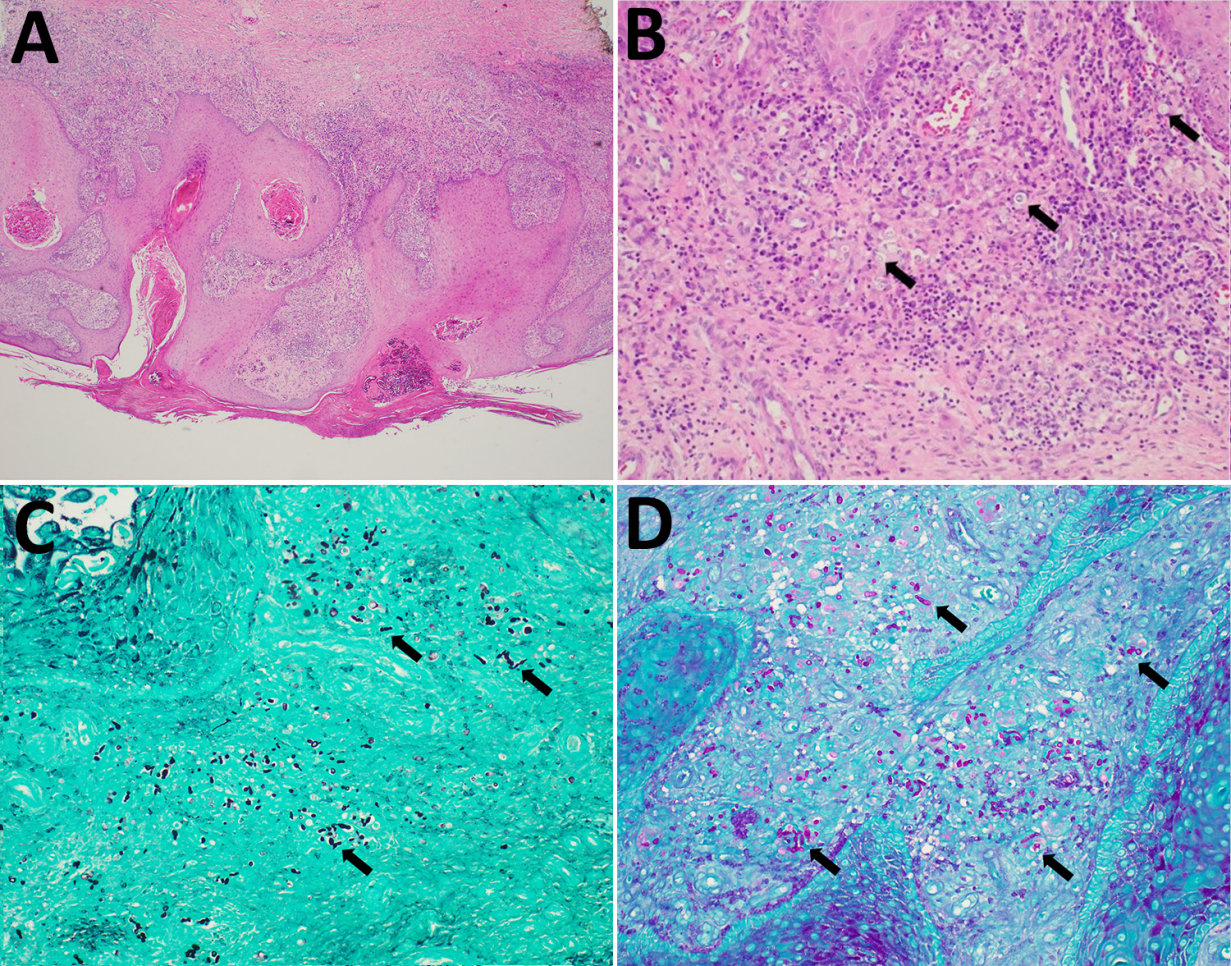
Histologic (A, B) and histochemical (C, D) findings from case of cutaneous *Paraconiothyrium cyclothyrioides* in lung transplant recipient, Georgia, USA. A) Skin biopsy from right shin revealed psuedoepitheliomatous hyperplasia of the epidermis overlying a mixed dermal inflammatory infiltrate. Hematoxylin and eosin stain; original magnification ×40. B) Polymorphic fungal elements (arrows) seen within the superficial dermis. Hematoxylin and eosin stain; original magnification ×200. C) Numerous fungal hyphal elements and yeast-like forms (arrows) highlighted within the dermis. Grocott methenamine silver stain; original magnification ×200. D) Fungal hyphal elements and yeast-like forms (arrows) noted by Periodic acid–Schiff stain; original magnification ×200.

Pending species identification, and under the supervision of an infectious diseases physician, the patient began oral posaconazole treatment. Treating physicians initially prescribed isavuconazole, but the treatment was cost prohibitive. The patient achieved therapeutic posaconazole levels (trough 1.8 µg/mL) and, after 6 weeks of treatment, the skin lesions healed, leaving 2 residual scars. His treatment course was complicated by tremor and insomnia related to drug-drug interactions between posaconazole and tacrolimus, despite appropriate tacrolimus dose reduction. Posaconazole was discontinued after 6 weeks, with clinical resolution.

Technicians ultimately identified dematiaceous mold from the patient’s culture, initially speciated by a reference laboratory as *Phanerochaete* species. Two months after the specimen was collected (8 months after the lesions first appeared and after treatment completion), DNA sequencing analysis revealed the mold to be *P. cyclothyrioides*. The isolate was unavailable for antifungal drug susceptibility testing.

This case of cutaneous *P. cyclothyrioides* in a lung transplant recipient highlights the pathogen as a potential cause of chronic, indolent skin infections in immunocompromised persons. Biopsy and culture are essential for diagnosing cutaneous infections in immunocompromised patients, and DNA sequencing is required to identify *P. cyclothyrioides*. Prompt referral to an infectious diseases specialist or a larger medical center with experience in transplantation can help promote accurate diagnosis and treatment while final speciation results are pending. If the biopsy or culture reveals fungus and species identification is delayed or not possible, healthcare providers should consider broad-spectrum antifungal therapies. The prolonged time needed for diagnosis, therapy initiation, and causative species identification in this case report aligns with previous reports (*3*–*8*), emphasizing the importance of heightened clinical suspicion. Further, the timeline of this case reinforces the need to develop rapid diagnostic tests to identify rare molds and distinguish between infection, colonization, and contamination (*1*,*2*).

In summary, our report underscores the need for heightened clinical awareness, improved diagnostics, and better antifungal options for rare mold infections such as *P*. *cyclothyrioides* and emphasizes the importance of ongoing surveillance. Treatment challenges associated with rare mold infections are common, as illustrated in this case, because appropriate antifungal drugs can be expensive, cause marked side effects, and interact with other drugs, including immunosuppressants frequently used by at-risk patients (*1*,*2*). In this instance, the patient’s infection resolved successfully with posaconazole. Prior *P. cyclothyrioides* case reports involved successful infection resolution following treatment with posaconazole, itraconazole, isavuconazole, or voriconazole (*3*,*5*–*9*).
